# Self-reported preoperative anxiety and depression associated with worse patient-reported outcomes for periacetabular osteotomy and hip arthroscopy surgery

**DOI:** 10.1093/jhps/hnae029

**Published:** 2024-08-23

**Authors:** Ann E Richey, Nicole Segovia, Katherine Hastings, Christian Klemt, Stephanie Y Pun

**Affiliations:** Department of Orthopaedic Surgery, Stanford University School of Medicine, 453 Quarry Road, Palo Alto, CA 94304, USA; Department of Orthopaedic Surgery, Stanford University School of Medicine, 453 Quarry Road, Palo Alto, CA 94304, USA; Department of Orthopaedic Surgery, Stanford University School of Medicine, 453 Quarry Road, Palo Alto, CA 94304, USA; Department of Orthopaedic Surgery, Stanford University School of Medicine, 453 Quarry Road, Palo Alto, CA 94304, USA; Department of Orthopaedic Surgery, Stanford University School of Medicine, 453 Quarry Road, Palo Alto, CA 94304, USA

## Abstract

Adverse mental health status has been linked to less successful surgical outcomes across several orthopaedic subspecialties. Mental health represents a modifiable risk factor that can be optimized preoperatively to maximize outcomes for hip preservation surgery. This study examines the relationship between preoperative mental health status and preoperative and postoperative outcomes for adolescent and adult patients undergoing hip preservation surgery. A prospectively enrolled registry of patients undergoing periacetabular osteotomy or hip arthroscopy at a single institution between 2013 and 2021 was retrospectively reviewed to collect demographics and outcomes before and after surgery. We identified patients self-reporting anxiety/depression or no anxiety/depression preoperatively based on responses to the EuroQol-5D anxiety/depression dimension and compared their preoperative and postoperative Hip disability and Osteoarthritis Outcome Scores (HOOSs) using multivariable linear models and multivariable mixed effects models. Seventy-three patients were included, 40 patients with no anxiety/depression and 33 patients with anxiety/depression. Patients with anxiety/depression had worse preoperative HOOS pain (*b* = −12.5, *P* = .029), function in daily living (*b* = −12.0, *P* = .045), function in sports and recreational activities (*b* = −15.1, *P* = .030), and quality of life (*b* = −16.3, *P* = .005) as compared to patients with no anxiety/depression. Patients with anxiety/depression had worse postoperative HOOS compared to patients with no anxiety/depression, but these associations were not statistically significant after adjusting for preoperative HOOS. There were no significant differences between both groups for percent achieving minimal clinically important difference. Patients who reported anxiety/depression preoperatively had worse preoperative pain and function before hip preservation surgery, with both groups achieving similar levels of clinical effectiveness.

## Introduction

Recent studies have illustrated the potential of integrated care models to significantly increase the quality of care delivered to orthopaedic patients [1]. While orthopaedic surgeons may notice adverse psychosocial factors with their patients, some may still avoid referrals for mental health treatment due to time constraints or stigma [2]. This may occur despite the awareness that mental health status represents a modifiable risk factor and thus can potentially be optimized before and after surgery in order to maximize patient outcomes [3, 4]. Therefore, moving towards an integrated approach in medical care, and specifically in orthopaedic surgery, can increase the quality of patient care and potentially surgical outcomes.

Hip preservation surgery, including periacetabular osteotomy (PAO) and hip arthroscopy, aims to prevent or delay the onset of hip arthritis and thus the subsequent need for hip replacement. Therefore, hip preservation surgery is commonly performed in adolescent and young adult patients who are usually highly independent and otherwise healthy. After surgery, patients have limited weight bearing for a few weeks to months, are much more dependent on caregivers for help with activities of daily living, and are usually limited in function for 6 months or more before being able to return to sports. These restrictions may cause young patients to feel a loss of independence, isolation from peers, and loss of identity due to inability to participate in their sport. The young patient age makes them particularly vulnerable to mental health problems, with prior research reporting psychological care needs during and after hip preservation surgery [5]. Assessing and understanding a patient’s psychological care needs before hip preservation surgery could further improve mental and physical outcomes after surgery.

Prior studies in the orthopaedic literature demonstrate that patient outcomes vary due to a variety of modifiable and nonmodifiable patient and surgical factors. Recent studies demonstrated that poor mental health of patients is associated with inferior functional and clinical outcomes following orthopaedic interventions [6–11]. These prior works showed that poor mental health status significantly correlated with increased pain scores, increased recovery periods, poorer functional outcomes, as well as reduced patient satisfaction [6–11]. In particular, poor preoperative mental health scores in adolescent patients undergoing hip preservation surgery were predictive of elevated postoperative pain scores [7]. Recently, the initial recovery after carpal tunnel release was reported to be related solely to the symptoms of anxiety and not to the severity of the neuropathy, highlighting the adverse effect that preoperative mental health may have on postoperative patient outcomes [6].

It remains unclear whether preoperative anxiety and depression predict postoperative functional outcomes of hip preservation surgery in adolescent and young adult patients. The purpose of this study was to investigate whether the Euro-Qol-5D (EQ-5D) anxiety/depression dimension, which assesses the presence of anxiety or depression, can predict patient-reported pain and function, as measured by the Hip disability and Osteoarthritis Outcome Score (HOOS) questionnaire, before and after PAO and hip arthroscopy surgery. We hypothesize that patients reporting anxiety/depression preoperatively would report inferior postoperative pain and function scores.

## Materials and methods

### Study population

**Figure 1.**
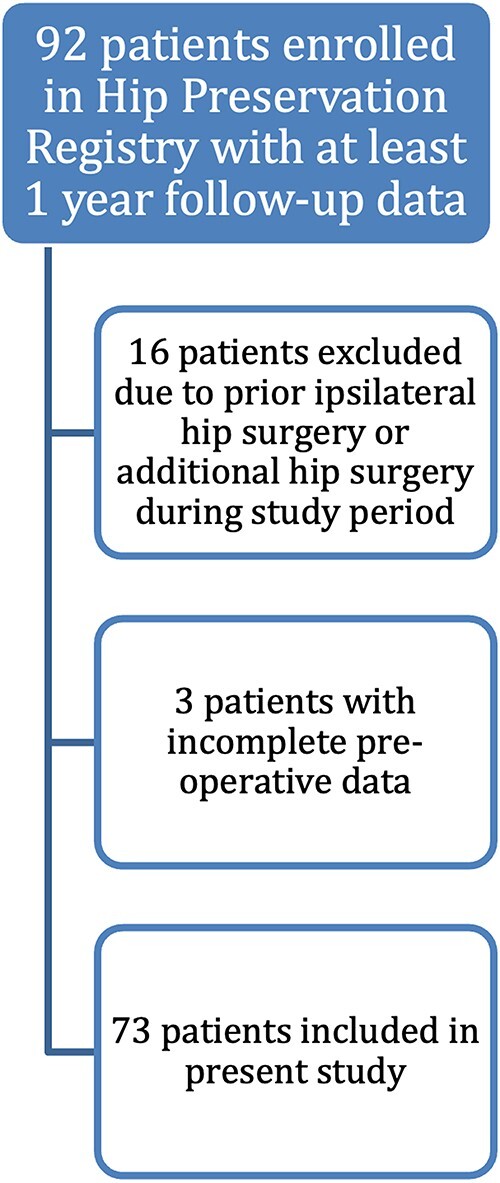
Inclusion and exclusion criteria for analysis.

After obtaining Institutional Review Board approval, we retrospective reviewed patient-reported outcome (PRO) measures that were prospectively collected from patients at a single institution undergoing either PAO or hip arthroscopy surgery from 2013 to 2021. Patients were included in the analysis if they had complete preoperative and postoperative PROs with at least 1-year postoperative follow-up. Patients with prior ipsilateral hip surgery or bilateral hip surgery during the study duration were excluded from analysis to minimize potential confounding indicated in prior literature [12]. (Fig. 1)

### Study procedures

Patients were consented for the research study at their preoperative clinical visit, and all patients completed a series of PROs questionnaires at the preoperative clinical visit and each annual postoperative clinical visit. If patients were not present in person for the annual postoperative clinical visit, the PRO questionnaires were sent to the patient’s electronic mailing address to complete.

### PRO measurements

We utilized the EQ-5D anxiety/depression dimension to assess patient’s mental health status, as well as the HOOS questionnaire to evaluate functional outcomes [13, 14]. The PRO questionnaires (EQ-5D and HOOS) were collected preoperatively within 1 month prior to the hip surgery at their preoperative clinical appointment. The PRO questionnaires were then collected annually postoperatively. The EQ-5D anxiety/depression dimension categorizes mental health status into three levels: ‘(i) I am not anxious or depressed, (ii) I am moderately anxious or depressed, and (iii) I am extremely anxious or depressed’. The HOOS questionnaire yields outcome scores for symptom, pain, function in daily living, function in sports and recreational activities, and quality of life. Scores range from 0 (extreme symptoms) to 100 (no symptoms). The minimal clinically important difference (MCID) values were derived from the studies by Wasko *et al*. based on their calculations for PAO patients and Kemp *et al*. based on their calculations for hip arthroscopy patients [15, 16]. For HOOS symptoms, we were looking for a delta of at least 10.3 for PAO patients and 9 for hip arthroscopy patients; for HOOS pain, we were looking for a delta of at least 10.2 for PAO patients and 9 for hip arthroscopy patients; for HOOS daily living, we were looking for a delta of at least 10.8 for PAO patients and 6 for hip arthroscopy patients; for HOOS sports/recreational activities, we were looking for a delta of at least 12.6 for PAO patients and 10 for hip arthroscopy patients; for HOOS quality of life, we were looking for a delta of at least 11.2 for PAO patients and 11 for hip arthroscopy patients [15, 16].

### Statistical analysis

For the analysis, patients were divided into two groups based on their preoperative EQ-5D assessment. Patients who selected ‘“I am not anxious or depressed” preoperatively’ were in the no anxiety/depression group, and patients who selected ‘I am moderately anxious or depressed’ or ‘I am extremely anxious or depressed’ preoperatively were in the anxiety/depression group. Preoperative HOOS from the no anxiety/depression and the anxiety/depression groups were compared using multivariable linear models adjusting for age and procedure. Postoperative HOOSs from the no anxiety/depression and the anxiety/depression groups were compared using the multivariable mixed effects model, adjusting for multiple follow-ups per person. Other adjustment variables included age, procedure (PAO or hip arthroscopy), follow-up time, and preoperative HOOS. All data analyses were performed using RStudio version 1.1.456 (Boston, MA), using a two-sided level of significance at *P* = .05.

## Results

### Patient population

Based on a power analysis, a sample size of 66 provides at least 80% power to detect a 25% difference between groups (no anxiety/depression and anxiety/depression), with an extra 10% to account for loss to follow-up. A total of 73 patients (56 females; 17 males) with complete pre- and postoperative HOOSs were included for analysis. This includes 40 patients with no anxiety/depression and 33 patients with anxiety/depression. The average age of the patient cohort was 24.8 years (range: 10.2–58.5), and the average follow-up time from surgery was 2.1 years (range: 1.0–6.1). Patient demographics and surgical parameters for both study cohorts are summarized in Table 1.

**Table 1.** Patient demographics and characteristics.

Patient characteristics	Total (*n* = 73)	No anxiety/depression (*n* = 40)	Anxiety/depression (*n* = 33)
Gender, count (%)			
Male	17 (23.3)	11 (27.5)	6 (18.2)
Female	56 (76.7)	29 (72.5)	27 (81.8)
Age, years			
Mean (range)	24.8 (10.2–58.5)	20.8 (10.2–41.2)	29.7 (13.4–58.5)
Ethnicity, count (%)			
Hispanic/Latino	18 (24.7)	10 (25.0)	8 (24.2)
Not Hispanic/Latino	55 (75.3)	30 (75.0)	25 (75.8)
Race, count (%)			
White	50 (68.5)	24 (60.0)	26 (78.8)
Black or African American	1 (1.4)	0 (0.0)	1 (3.0)
Asian	6 (8.2)	4 (10.0)	2 (6.1)
American Indian or Alaska Native	1 (1.4)	0 (0.0)	1 (3.0)
Other	15 (20.5)	12 (30.0)	3 (9.1)
Procedure, count (%)			
PAO	52 (71.2)	32 (80.0)	20 (60.6)
Hip arthroscopy	21 (28.8)	8 (20.0)	13 (39.4)
Follow-up (years)			
Mean (range)	2.1 (1.0–6.1)	2.1 (1.0–5.8)	2.1 (1.0–6.1)
Surgery side, count (%)			
Right	36 (49.3)	16 (40.0)	20 (60.6)
Left	37 (50.7)	24 (60.0)	13 (39.4)

### Preoperative EQ-5D and preoperative HOOSs

Compared to patients with no anxiety/depression, patients with anxiety/depression had worse preoperative HOOS pain (β = −12.5, *P* = .029), function in daily living (β = −12.0, *P* = .045), function in sports and recreation activities (β = −15.1, *P* = .030), and quality of life (β = −16.3, *P* = .005; Table 2). There were no significant differences between patients with no anxiety/depression and patients with anxiety/depression for preoperative HOOS symptom (Table 2).

**Table 2.** Preoperative HOOS for patients with no anxiety/depression vs. anxiety/depression.

HOOS	Estimate (CI)	*P*-value
Symptom	−10.3 (−21.5, 0.8)	0.068
Pain	−12.5 (−23.7, −1.3)	0.029*
Daily living	−12.0 (−23.8, −0.3)	0.045*
Sports and recreation	−15.1 (−28.7, −1.5)	0.030*
Quality of life	−16.3 (−27.3, −5.2)	0.005*

Model adjusted for age and procedure.

### Preoperative EQ-5D and postoperative HOOS

Adjusting for preoperative HOOSs, age, procedure, and follow-up time, compared to patients with no anxiety/depression, patients with anxiety/depression had worse postoperative HOOS symptom, pain, daily living, sports and recreation, and quality of life; however, these differences were not statistically significant (Table 3). Although postoperative HOOSs were not significantly different between patients with and without anxiety/depression after adjusting for preoperative scores, age, procedure, and follow-up time, average HOOS subscale scores for patients with anxiety/depression ranged from 0.3 to <11.0 scores for patients without anxiety/depression with a large amount of variability.

**Table 3.** Postoperative HOOS for patients with no anxiety/depression vs. anxiety/depression.

HOOS	Estimate (confidence interval)	*P*-value
Symptom	−0.3 (−9.2, 8.6)	0.943
Pain	−3.4 (−10.5, 3.7)	0.364
Daily living	−4.1 (−10.7, 2.4)	0.234
Sports and recreation	−6.4 (−17.0, 4.1)	0.250
Quality of life	−11.0 (−24.1, 2.1)	0.112

Model adjusted for preoperative HOOS, age, procedure, and follow-up time.

### MCIDs

For PAO patients, there were no significant differences between the proportion of those who achieved MCID for patients with no anxiety/depression and patients with anxiety/depression (Table 4). Likewise, for hip arthroscopy patients, there were no significant differences between the proportion of those who achieved MCID for patients with no anxiety/depression and patients with anxiety/depression (Table 4). When looking at the patient population as a whole and adjusting for preoperative scores, procedure, age, and follow-up time, patients with anxiety/depression had lower odds of achieving MCID for HOOS pain compared to patients with no anxiety/depression (Odds Ratio = 0.06, *P* = .037, Table 5). For patients with anxiety/depression, the patients who reported extreme anxiety/depression (two out of two patients) preoperatively did not achieve MCID in HOOS symptom, pain, function in daily living, and function in sports and recreational activity scores.

**Table 4.** Patients in the no anxiety/depression and anxiety/depression groups who achieved MCID.

	No anxiety/depression		Anxiety/depression		
HOOS	Count	%	Count	%	*P*-value
PAO patients					
Symptom	16	50	14	70	0.258
Pain	23	72	16	80	0.742
Daily living	13	41	14	70	0.076
Sports and recreation	23	72	13	65	0.831
Quality of life	24	75	16	80	0.747
Hip arthroscopy patients					
Symptom	5	63	8	62	>0.999
Pain	7	88	9	69	0.607
Daily living	5	63	9	69	>0.999
Sports and recreation	6	75	11	85	0.618
Quality of life	6	75	8	62	0.656

**Table 5.** Preoperative EQ-5D anxiety/depression dimension vs. MCID achievement.^a^

HOOS	Odds ratio (95% confidence interval)	*P*-value
Symptom	0.76 (0.07, 8.55)	0.826
Pain	0.06 (0.01, 0.84)	0.037*
Daily living	1.45 (0.34, 6.30)	0.615
Sports and recreation	1.45 (0.34, 6.30)	0.133
Quality of life	0.47 (0.11, 2.00)	0.305

a Odds ratios are comparing anxiety/depression vs. no anxiety/depression, adjusting for preoperative scores, procedure, age, and follow-up time. PAO and hip arthroscopy patients were combined for this analysis as the sample size was limited to examine odds ratios separately although the analysis adjusted for surgery type to account for potential differences.

* Statistically significant at the alpha level, 0.05.

## Discussion

In our study, we found that poor preoperative mental health was associated with worse pain and worse functional outcome reports before and after hip surgery. Prior research has demonstrated that mental health influences surgical outcomes for orthopaedic patients in all specialties including spine, trauma, fractures, shoulder, sports medicine, hip, knee, and hand [9]. Our findings add to the body of knowledge that mental health reports do impact orthopaedic surgical outcomes and, in particular, hip preservation pain outcomes [6–11]. The findings from the present study add to the literature by examining PROs that extend beyond not only pain metrics but also function in daily living, function in sports and recreational activities, and quality of life preoperatively and postoperatively.

Our results suggest that poor mental health is associated with worse preoperative scores. In addition, a patient with a worse preoperative score may then have a worse postoperative score. Further research could be done to understand the relationship between the preoperative pain/functional outcome scores, postoperative pain/functional outcome scores, preoperative EQ-5D mental health dimension, and the postoperative EQ-5D mental health dimension to try and determine a cause-and-effect relationship. Furthermore, the observed difference in postoperative scores can help in patient counselling and expectation setting between the two groups.

### Minimum clinically important differences

Our results show that mental health factors might not necessarily be a barrier to meaningful change from preoperative to postoperative outcomes, since patients who reported anxiety/depression and no anxiety/depression achieved meaningful changes in HOOS domains. Our results align with some studies examining clinically relevant changes postoperatively for patients reporting a history of mental health disorders. Patients undergoing hip arthroscopy surgery who reported a history of mental health disorders had lower preoperative and postoperative PROs, but yielded clinically significant improvements [11]. However, other studies have found poor mental health to be linked with lower rates of achieving meaningful changes using other types of PROs for hip arthroscopy, demonstrating conflicting literature [17]. Future studies could further examine if extreme anxiety/depression is a barrier to meaningful changes postoperatively for patients undergoing hip preservation surgery.

### Clinical implications

Prior work has illustrated that an integrated treatment approach for adolescents, including the use of a psychologist, significantly improved pain, quality of life, and mental health symptoms after hip preservation surgery [7]. Our findings support further research in this area to determine integrated treatment approaches that may be helpful for both adolescents and adults with their pain and functional outcomes.

### Limitations

There are a few limitations that may warrant caution in the interpretation of findings. First, the present study investigated the effect of mental health status on PROs from a single institution. Therefore, there could be a potential for selection bias since the patients might be less representative of the general population of the USA. Socioeconomic differences may be associated with differences in access to healthcare resources that can mitigate mental health distress. Second, while similar studies on this research area utilized a comparable number of patients and our power calculation demonstrated that the statistical analysis is sufficiently powered, we did not have enough patients to perform stratified analyses and examine paediatric and adult outcomes separately [4, 5, 7, 18]. Although we adjusted for age, future research in this area should examine paediatric and adults separately as outcomes may differ. Third, the EQ-5D anxiety/depression dimension is based on self-reporting and has its limitations. Patients might have a different perspective for what is considered to be none, moderate, or extreme anxiety/depression. Self-reported data are subjective in nature and may be different across individuals and groups, especially with the range of ages in our patient population although this instrument has been used in adolescent and adult populations [19]. However, in addition to a 3-level version, the EQ-5D also has a 5-level version to measure health status [20]. The 3-level version was used to avoid survey fatigue since patients were completing multiple PRO measurements. However, future research could be performed with more granular instruments, such as the 5-level version, to further understand the association between mental health and surgical outcomes. Fourth, a recent study found that patients undergoing hip arthroscopy surgery who had minimal or mild depressive symptoms had statistically and clinically significant better outcomes than patients who had moderate to severe depressive symptoms [18]. Since our cohort only had two patients who reported extreme anxiety/depression, we were not able to compare moderate anxiety/depression to extreme anxiety/depression. Nonetheless, this represents a limitation noted in similar studies on this topic due to the range of mental health disorders and severity [11]. Fifth, recent literature has suggested that psychotropic medications may influence postoperative outcomes for patients undergoing hip arthroscopy surgery, but psychotropic medication use was unavailable in our data, so we were unable to include it in our adjustment model [21].

## Conclusion

Overall, compared to patients who reported no anxiety/depression before PAO or hip arthroscopy surgery, patients who reported moderate or extreme anxiety/depression before surgery did not have significantly worse self-reported pain and functional outcome scores before or after hip preservation surgery. Further research should be conducted to understand how to address psychosocial needs of patients who are undergoing hip preservation surgery, since there are differing results across studies regarding mental health outcomes and hip preservation surgical outcomes. With an integrated approach, we can potentially increase the quality of care and surgical outcomes for orthopaedic patients.

## Data Availability

The data underlying this article cannot be shared publicly due to privacy concerns for the patients who participated.

## References

[1] Hwang W, Chang J, Laclair M et al. Effects of integrated delivery system on cost and quality. *Am J Manag Care* 2013;19:e175–84. 23781916

[2] Vranceanu AM, Beks RB, Guitton TG et al. How do orthopaedic surgeons address psychological aspects of illness? *Arch Bone Jt Surg* 2017;5:2–9. 28271080 PMC5339350

[3] Kamalapathy P, Kurker KP, Althoff AD et al. The impact of mental illness on postoperative adverse outcomes after outpatient joint surgery. *J Arthroplasty* 2021;36:2734–41. doi: 10.1016/j.arth.2021.04.002 33896669

[4] Richard HM, Nguyen DC, Podeszwa DA et al. Perioperative interdisciplinary intervention contributes to improved outcomes of adolescents treated with hip preservation surgery. *J Pediatr Orthop* 2018;38:254–59. doi: 10.1097/BPO.0000000000000816 27328119

[5] Podeszwa DA, Richard HM, Nguyen DC et al. Preoperative psychological findings in adolescents undergoing hip preservation surgery. *J Pediatr Orthop* 2015;35:253–57. doi: 10.1097/BPO.0000000000000243 24992348

[6] Ryan C, Miner H, Ramachandran S et al. General anxiety is associated with problematic initial recovery after carpal tunnel release. *Clin Orthop* 2022;480:1576–81. doi: 10.1097/CORR.0000000000002115 35023866 PMC9278949

[7] Richard HM, Cerza SP, De La Rocha A et al. Preoperative mental health status is a significant predictor of postoperative outcomes in adolescents treated with hip preservation surgery. *J Child Orthop* 2020;14:259–65. doi: 10.1302/1863-2548.14.200013 32874357 PMC7453166

[8] Dumont GD, Land J, Battle NC et al. Factors associated with high pain catastrophizing in patients undergoing hip arthroscopy for femoroacetabular impingement syndrome. *J Hip Preserv Surg* 2020;7:483–86. doi: 10.1093/jhps/hnaa034 33948203 PMC8081422

[9] Ayers DC, Franklin PD, Ring DC. The role of emotional health in functional outcomes after orthopaedic surgery: extending the biopsychosocial model to orthopaedics. *J Bone Joint Surg Am* 2013;95:e165. doi: 10.2106/JBJS.L.00799 PMC3808180 24196477

[10] Rolfson O, Dahlberg LE, Nilsson JA et al. Variables determining outcome in total hip replacement surgery. *J Bone Joint Surg Br* 2009;91:157–61. doi: 10.1302/0301-620X.91B2.20765 19190046

[11] Lansdown DA, Ukwuani G, Kuhns B et al. Self-reported mental disorders negatively influence surgical outcomes after arthroscopic treatment of femoroacetabular impingement. *Orthop J Sports Med* 2018;6:2325967118773312. doi: 10.1177/2325967118773312 PMC5960865 29796402

[12] Pun SY, Hosseinzadeh S, Dastjerdi R et al. What are the early outcomes of true reverse periacetabular osteotomy for symptomatic hip overcoverage?. *Clin Orthop* 2021;479:1081–93. doi: 10.1097/CORR.0000000000001549 33296152 PMC8052029

[13] Lyman S, Lee YY, Franklin PD et al.. Validation of the HOOS, JR: a short-form hip replacement survey. *Clin Orthop* 2016;474:1472–82. doi: 10.1007/s11999-016-4718-2 26926772 PMC4868170

[14] EQ-5D-3L . EuroQol. https://euroqol.org/information-and-support/euroqol-instruments/eq-5d-3l/ (22 May 2024, date last accessed).

[15] Wasko MK, Yanik EL, Pascual-Garrido C et al. Psychometric properties of patient-reported outcome measures for periacetabular osteotomy. *J Bone Joint Surg Am* 2019;101:e21. doi: 10.2106/JBJS.18.00185 30893237

[16] Kemp JL, Collins NJ, Roos EM et al. Psychometric properties of patient-reported outcome measures for hip arthroscopic surgery. *Am J Sports Med* 2013;41:2065–73. doi: 10.1177/0363546513494173 23835268

[17] Hall A, Dandu N, Sonnier JH et al. The influence of psychosocial factors on hip surgical disorders and outcomes after hip arthroscopy: a systematic review. *Arthrosc J Arthrosc Relat Surg Off Publ Arthrosc Assoc N Am Int Arthrosc Assoc* 2022;38:3194–206. doi: 10.1016/j.arthro.2022.05.003 35660519

[18] Sochacki KR, Brown L, Cenkus K et al. Preoperative depression is negatively associated with function and predicts poorer outcomes after hip arthroscopy for femoroacetabular impingement. *Arthrosc J Arthrosc Relat Surg Off Publ Arthrosc Assoc N Am Int Arthrosc Assoc* 2018;34:2368–74. doi: 10.1016/j.arthro.2018.03.020 29789247

[19] Brooks R . EuroQol: the current state of play. *Health Policy* 1996;37:53–72. doi: 10.1016/0168-8510(96)00822-6 10158943

[20] Bilbao A, García-Pérez L, Arenaza JC et al. Psychometric properties of the EQ-5D-5L in patients with hip or knee osteoarthritis: reliability, validity and responsiveness. *Qual Life Res* 2018;27:2897–908. doi: 10.1007/s11136-018-1929-x 29978346

[21] Philippon MJ, Patterson DC, Briggs KK. Hip arthroscopy and femoroacetabular impingement in the pediatric patient. *J Pediatr Orthop* 2013;33:S126–130. doi: 10.1097/BPO.0b013e318274f834 23764784

